# Supplementary material for: Glacial isostatic adjustment reduces past and future Arctic subsea permafrost
Source: Nat Commun. 2024 Apr 15;15:3232. doi: 10.1038/s41467-024-45906-8 (PMC11018745; doi:10.1038/s41467-024-45906-8)
Supplement: Supplementary file 1 — Supplementary Information [file 41467_2024_45906_MOESM1_ESM.pdf]

Supporting Information for: Glacial Isostatic Adjustment reduces past and future Arctic subsea permafrost

Roger C. Creel*[1,2], Frederieke Miesner[3], Stiig Wilkenskjeld[4], Jacqueline Austermann[2], and Pier Paul Overduin[3]

1. Department of Physical Oceanography, Woods Hole Oceanographic Institution, Woods Hole, Massachusetts, USA
2. Lamont-Doherty Earth Observatory, Columbia University, New York, USA.
3. Alfred Wegener Institute Helmholtz-Centre for Polar and Marine Research, Potsdam, Germany
4. Max Planck Institute for Meteorology, Hamburg, Germany.

*roger.creel@whoi.edu

Contents of this file

1. Table S1 & S2, Figures S1 to S4

Model	Ice History	Viscosity	GMSL curve	GIA	air temp.	seabed temp.
Legacy	CLIMBER2	n/a	Grant	No	CLIMBER2	Dmitrenko
Base	ICE-6G	n/a	Waelbroeck	No	CLIMBER2	Dmitrenko
GIA	ICE-6G	VM5a	Waelbroeck	Yes	CLIMBER2	Dmitrenko
GIA-2	ICE-6G	l71.ump3.lm50	Waelbroeck	Yes	CLIMBER2	Dmitrenko
GIA-3	ANU-6G	l71.ump3.lm50	Waelbroeck	Yes	CLIMBER2	Dmitrenko

Table S1: **Model reference names and metadata.** Model reference names with associated ice sheet histories, solid Earth structures, global mean sea level (GMSL) curves, air and seabed temperatures, and whether that model includes glacial isostatic adjustment (GIA). VM5a refers to the VM5a viscosity structure of [1]. The l71.ump3.lm50 viscosity structure has a lithospheric thickness of 71 km, an upper mantle viscosity of 0.3 x 10^{21} Pa s, and a lower mantle viscosity of 50 x 10^{21} Pa s. 'Grant' refers to [2]; 'Waelbroeck' refers to [3]; 'Dmitrenko' refers to [4]. See Methods for details of ice sheet history construction.

#	exp_id	Scenario	GCM forcing
5/6	exp05/exp06	RCP8.5	NorESM1-M
6/5	exp06/exp05	RCP8.5	MIROC-ESM-CHEM/MIROC5
7/7	exp07/exp07	RCP2.6	NorESM1-M/MIROC5
8/3	exp08/exp03	RCP8.5	CCSM4/ACCESS1p3
9/9	exp09/exp09	RCP8.5	NorESM1-M/MIROC5
10/10	exp10/exp10	RCP8.5	NorESM1-M/MIROC5
12/b01	exp12/expb01	RCP8.5	CCSM4/CNRM-CM6-1
13/6	exp13/exp06	RCP8.5	NorESM1-M
A5/8	expA5/exp08	RCP8.5	HadGEM2-ES
A6/8	expA6/exp08	RCP8.5	CSIRO-Mk3.6.0/HadGEM-ES
A7/a01	expA7/expa01	RCP8.5	IPSL-CM5A-MR
A8/07	expA8/exp07	RCP2.6	IPSL-CM5A-MR/ACCESS1p3
B6/b02	expB6/expb02	SSP5-8.5	CNRM-CM6-1
B7/b05	expB7/b05	SSP1-2.6	CNRM-CM6-1/CNRM-EMS2-1
B8/b03	expB8/expb03	SSP5-8.5	UKESM1-0-LL
B9/b04	expB9/expb04	SSP5-8.5	CESM2
B10/b05	expB10/expb05	SSP5-8.5	CNRM-ESM2-1

Table S2: **Pairings of extended Greenland and Antarctic future climate experiments used in ISMIP-6 ice models [5].** Generalized Circulation Model (GCM) forcing cells with (without) a slash represent dissimilar (identical) Greenland/Antarctic forcing.

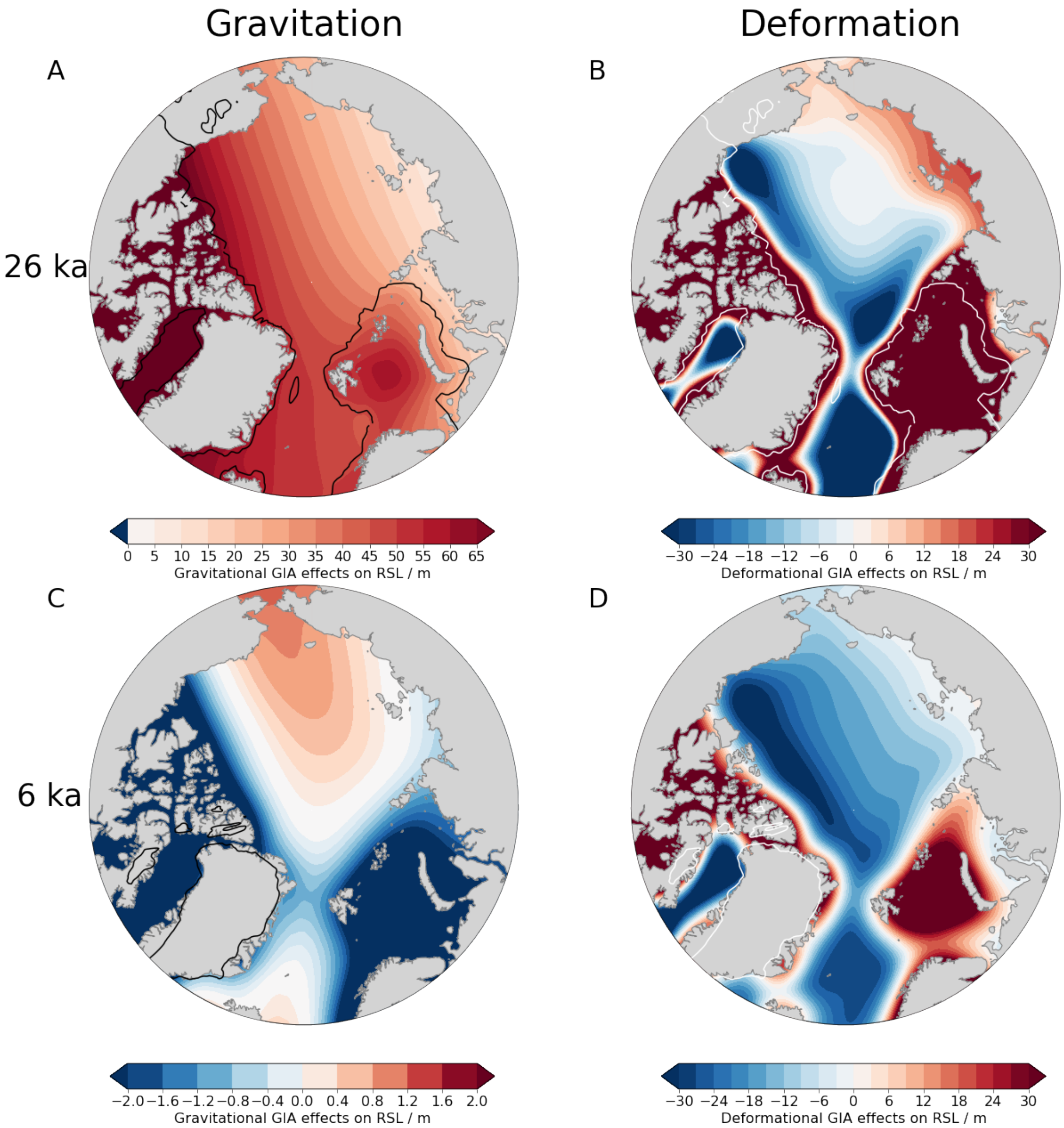

Figure S1: **Gravitational and deformational effects of glacial isostatic adjustment on relative sea level (RSL) at Last Glacial Maximum (26 ka) and the mid-Holocene (6 ka). (A/C)** Gravitational effects, composed of the gravitational effects associated with solid-Earth deformation and the self-gravitation of ice sheets at 26 (A) and 6 (C) ka. (B) Deformational effects, composed of the deformational effect of water mass change on the solid Earth plus the uniform increase in sea level due to the addition of water, minus global mean sea level at 26 (A) and 6 (C) ka. Black (A/C) and white (B/D) outlines denote the margins of the northern hemisphere ice sheets during the Last Glacial Maximum and mid-Holocene, respectively, following [1].

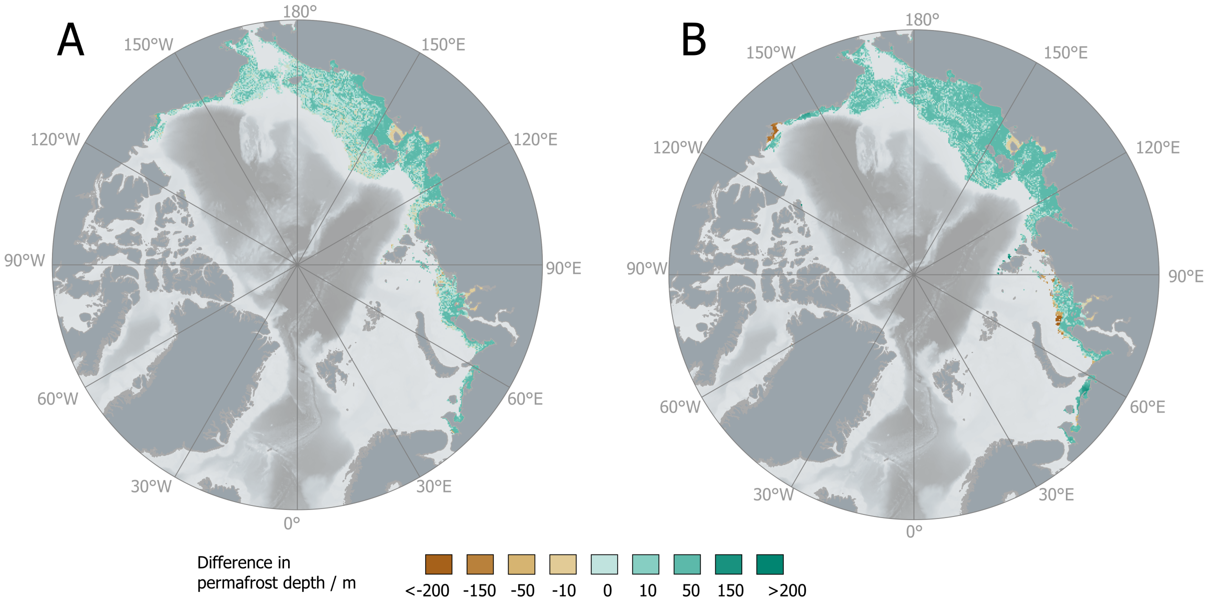

Figure S2: **Comparison of subsea permafrost thickness between glacial isostatic adjustment (GIA) run and sensitivity tests.** (A) Difference in subsea permafrost thickness at 1850 between the GIA and GIA-2 model run, which shows the effect of a different solid Earth structure. (B) Same as (A), but for the GIA-3 model run, showing the effect of a different Northern Hemisphere ice history in addition to the solid Earth structure in (A). 70% of the area in GIA-2 and GIA-3 deviates from the GIA run by less than 20m 25m, respectively. See Table S1 and Methods for details about the differences between the GIA, GIA-2, and GIA-3 models.

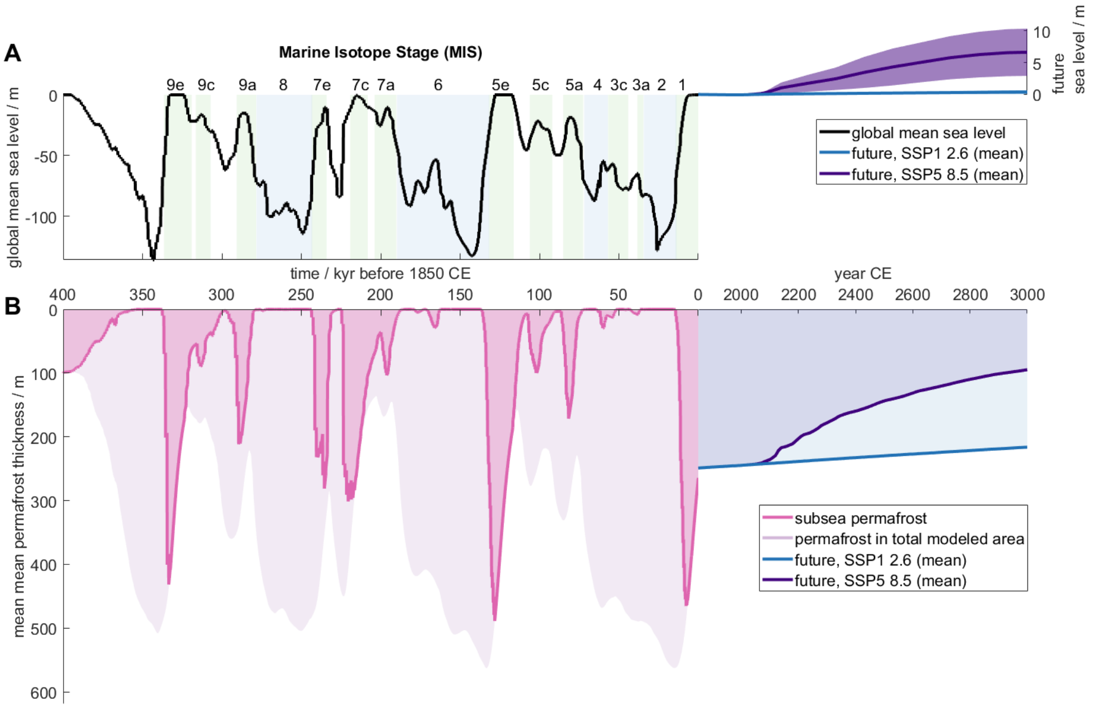

Figure S3: **Subsea permafrost thickness as a function of time between 400 kyr BP and 1850 CE and projected until 3000 CE.** (A) Past and projected future global mean sea level. (B) Subsea permafrost thickness from the base run for all permafrost within the model region (light pink) and under the Arctic Ocean (dark pink) are shown for the past 400 kyr and projected until 3000 CE. See Fig. 1 for more information.

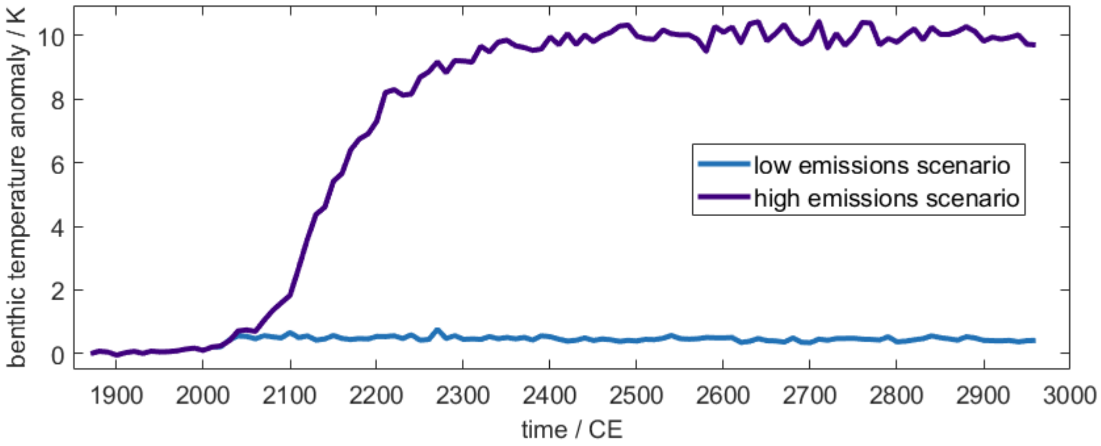

Figure S4: **Projected benthic temperature anomaly applied to Arctic bottom waters for 1900 to 3000 CE.** Benthic temperature anomaly is adopted following [6] for low emissions (SSP1-2.6, blue line) and high emissions (SSP5-8.5, purple line) climate scenarios.

Supplementary References

[1] Peltier, W. R., Argus, D. F. & Drummond, R. Space geodesy constrains ice age terminal deglaciation: The global ICE-6G_C (VM5a) model. *Journal of Geophysical Research: Solid Earth* **120**, 450–487 (2015).

[2] Grant, K. M. *et al.* Sea-level variability over five glacial cycles. *Nature Communications* **5**, 5076 (2014).

[3] Waelbroeck, C. *et al.* Sea-level and deep water temperature changes derived from benthic foraminifera isotopic records. *Quaternary Science Reviews* **21**, 295–305 (2002).

[4] Dmitrenko, I. A. *et al.* Recent changes in shelf hydrography in the Siberian Arctic: Potential for subsea permafrost instability. *Journal of Geophysical Research: Oceans* **116** (2011).

[5] Nowicki, S. *et al.* Experimental protocol for sea level projections from ISMIP6 stand-alone ice sheet models. *The Cryosphere* **14**, 2331–2368 (2020).

[6] Wilkenskjeld, S., Miesner, F., Overduin, P. P., Puglini, M. & Brovkin, V. Strong increase in thawing of subsea permafrost in the 22nd century caused by anthropogenic climate change. *The Cryosphere* **16**, 1057–1069 (2022). URL `https://tc.copernicus.org/articles/16/1057/2022/`. Publisher: Copernicus GmbH.
